# Supplementary material for: Framework for a High-Throughput Screening Method to Assess Polymer/Plasticizer Miscibility: The Case of Hydrocarbons in Polyolefins
Source: Macromolecules. 2024 May 14;57(10):4637–47. doi: 10.1021/acs.macromol.3c01764 (PMC11140736; doi:10.1021/acs.macromol.3c01764)
Supplement: Supplementary file 1 — ma3c01764_si_001.pdf [file ma3c01764_si_001.pdf]

Supporting Information for

A Framework for a High Throughput Screening Method to Assess Polymer/Plasticizer Miscibility: The Case of Hydrocarbons in Polyolefins

*Lois Smith[a], Hossein Ali Karimi-Varzaneh[b], Sebastian Finger[b], Giuliana Giunta,[a,c] Alessandro Troisi[d], Paola Carbone[*a]*

[a] *Department of Chemical Engineering, School of Engineering, The University of Manchester, Oxford Road, M13 9PL, Manchester, United Kingdom*

[b]*Continental Reifen Deutschland GmbH, Jädekamp 30, D-30419 Hanover, Germany*

[c]*BASF, Carl-Bosch-Strasse 38, 67056, Ludwigshafen, Germany*

[d]*Department of Chemistry, Department of Chemistry, Crown Street, L69 7ZD, Liverpool, United Kingdom*

[*]*paola.carbone@manchester.ac.uk*

List of Systems Simulated and Miscibility Parameters

Table S1 List of all PL topologies simulated in this work, number of PL molecules in a system with 72 PI chains of molecular weight 300, the simulation box size after NPT equilibration and corresponding miscibility parameter, ζ. Error estimates were obtained using the standard error of the data.

Topology Number	Code	PL Molecule Number	Box Size (nm)	Miscibility Parameter, ζ
1	10B-3S-0.2-r	68	$8.654 \times 8.654 \times 25.229$	1.27 ± 0.004
2	10B-3S-0.3-r	57	$8.656 \times 8.656 \times 25.236$	1.32 ± 0.003
3	10B-3S-0.4-r	49	$8.656 \times 8.656 \times 25.235$	1.46 ± 0.01
4	10B-3S-0.5-r	43	$8.653 \times 8.653 \times 25.227$	1.40 ± 0.005
5	10B-3S-0.2-f	68	$8.654 \times 8.654 \times 25.229$	1.25 ± 0.003
6	10B-3S-0.3-f	57	$8.656 \times 8.656 \times 25.240$	1.32 ± 0.005
7	10B-3S-0.4-f	49	$8.656 \times 8.656 \times 25.235$	1.33 ± 0.004
8	10B-3S-0.5-f	43	$8.653 \times 8.653 \times 25.227$	1.39 ± 0.005
9	10B-4S-0.2-r	60	$8.655 \times 8.655 \times 25.233$	1.28 ± 0.003
10	10B-4S-0.3-r	49	$8.655 \times 8.655 \times 25.233$	1.43 ± 0.01
11	10B-4S-0.4-r	42	$8.657 \times 8.657 \times 25.240$	1.47 ± 0.01
12	10B-4S-0.5-r	36	$8.654 \times 8.654 \times 25.231$	1.75 ± 0.01
13	10B-4S-0.2-f	60	$8.655 \times 8.655 \times 25.233$	1.28 ± 0.004

14	10B-4S-0.3-f	49	8.655 × 8.655 × 25.233	1.34 ± 0.004
15	10B-4S-0.4-f	42	8.657 × 8.657 × 25.240	1.39 ± 0.004
16	10B-4S-0.5-f	36	8.654 × 8.654 × 25.231	1.47 ± 0.004
17	10B-5S-0.2-r	54	8.735 × 8.735 × 24.884	1.39 ± 0.01
18	10B-5S-0.3-r	43	8.143 × 8.143 × 28.490	1.56 ± 0.01
19	10B-5S-0.4-r	36	8.720 × 8.720 × 24.841	1.68 ± 0.01
20	10B-5S-0.5-r	31	8.720 × 8.720 × 24.839	48.25 ± 0.1
21	10B-5S-0.2-f	54	8.735 × 8.735 × 24.884	1.32 ± 0.005
22	10B-5S-0.3-f	43	8.143 × 8.143 × 28.490	1.39 ± 0.01
23	10B-5S-0.4-f	36	8.720 × 8.720 × 24.841	1.50 ± 0.01
24	10B-5S-0.5-f	31	8.720 × 8.720 × 24.839	1.62 ± 0.01
25	10B-6S-0.2-r	49	8.724 × 8.724 × 24.851	1.57 ± 0.01
26	10B-6S-0.3-r	39	8.653 × 8.653 × 25.228	1.75 ± 0.01
27	10B-6S-0.4-r	32	8.659 × 8.659 × 25.244	39.44 ± 0.21
28	10B-6S-0.5-r	27	8.652 × 8.652 × 25.226	69.87 ± 0.20
29	10B-6S-0.2-f	49	8.724 × 8.724 × 24.851	1.28 ± 0.005
30	10B-6S-0.3-f	39	8.653 × 8.653 × 25.228	1.43 ± 0.01
31	10B-6S-0.4-f	32	8.659 × 8.659 × 25.244	1.54 ± 0.01
32	10B-6S-0.5-f	27	8.652 × 8.652 × 25.226	1.72 ± 0.01
33	10B-7S-0.2-r	45	8.654 × 8.654 × 25.230	19.43 ± 0.04

34	10B-7S-0.3-r	35	8.653 × 8.653 × 25.229	25.23 ± 0.1
35	10B-7S-0.4-r	28	8.720 × 8.720 × 24.839	16.60 ± 0.02
36	10B-7S-0.5-r	24	8.654 × 8.654 × 25.229	17.04 ± 0.01
37	10B-7S-0.2-f	45	8.654 × 8.654 × 25.230	1.38 ± 0.01
38	10B-7S-0.3-f	35	8.654 × 8.654 × 25.230	1.51 ± 0.01
39	10B-7S-0.4-f	28	8.720 × 8.720 × 24.839	1.64 ± 0.01
40	10B-7S-0.5-f	24	8.654 × 8.654 × 25.229	1.68 ± 0.01
41	10B-8S-0.2-r	42	8.144 × 8.144 × 28.493	12.80 ± 0.03
42	10B-8S-0.3-r	32	8.145 × 8.145 × 28.495	64.83 ± 0.21
43	10B-8S-0.4-r	26	8.145 × 8.145 × 28.494	15.66 ± 0.01
44	10B-8S-0.5-r	22	8.143 × 8.143 × 28.490	37.16 ± 0.08
45	10B-9S-0.2-r	39	8.145 × 8.145 × 28.495	11.86 ± 0.02
46	10B-9S-0.3-r	29	8.143 × 8.143 × 28.490	79.41 ± 0.31
47	10B-9S-0.4-r	23	8.140 × 8.140 × 28.477	31.78 ± 0.11
48	10B-9S-0.5-r	20	8.146 × 8.146 × 28.499	10.77 ± 0.02

Vacuum Simulation Validation

In order to confirm the validity of calculating PL molecule square radius of gyration, R_g^2, acylindricity, c, and configurational entropy, S_{config}, with a simulation of a single PL in vacuum, we compared these analyses to those done from a system of a PL immersed in a PI melt. To do

so, we chose a small subset of 8 PLs, composed of both flexible and rod-like molecules. Fig. S3 and S4 display the results. All results are extracted over approximately 3 μs and the PL/PI simulations undergo an initial NPT equilibration, with the simulation parameters disclosed in the main text, to achieve an appropriate density. To ensure the PI melt is then properly equilibrated, a further 800 ns NVT simulation is performed which is sufficient time for the PI mean square internal distance (MSID) to plateau.

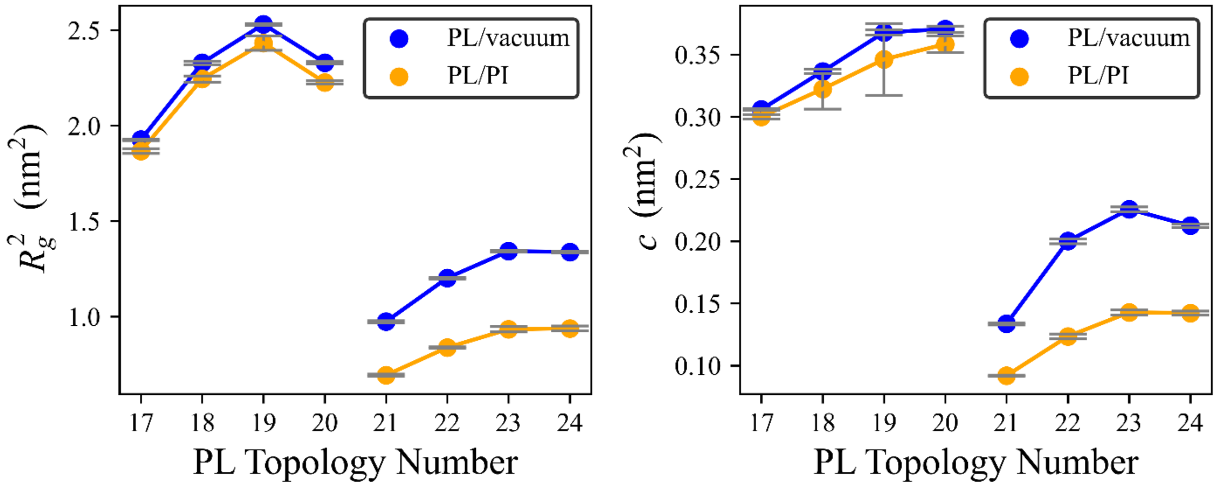

Figure S1: R_g^2 (left) and c (right) for 8 PL topologies as labelled. Values are taken from (blue) a PL molecule in an empty box and (orange) a PL molecule in an equilibrated PI melt. All results are extracted over a simulation of length 2 μs. The error bars are obtained with the standard error of block averages.

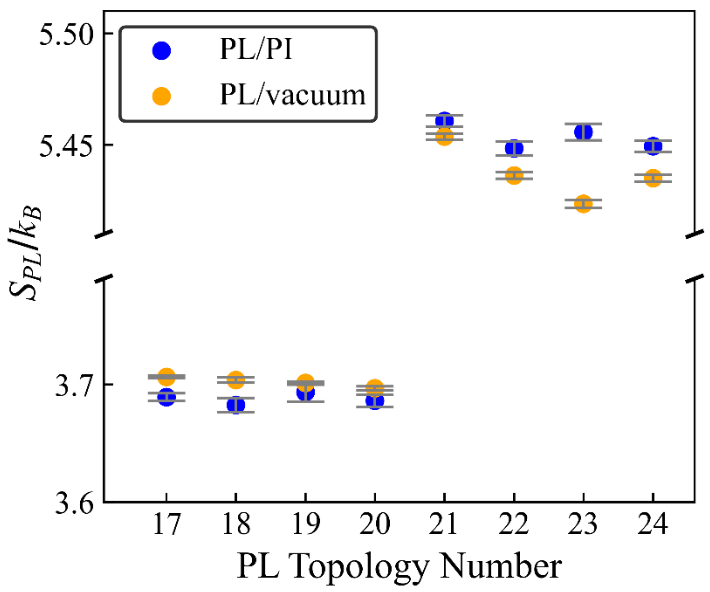

Figure S2: Configurational entropy, S_{PL}, for 8 PL topologies as labelled. Values are taken from (blue) a PL molecule in an empty box and (orange) a PL molecule in an equilibrated PI melt. All results are extracted over a simulation of length 2 μs. The error bars are obtained with the standard error of the data.

Figure S1 displays a comparison between (a) R_g^2 and (b) c in vacuum and in a PI melt. In both cases, a more significant difference is seen between the systems with flexible PLs (topologies 21, 22, 23 and 24), however the trend is similar. This is likely due to a swelling of the flexible PL molecules in vacuum, which is observed to a lesser extent in the rod-like molecules due to the rigid nature of their bond angles. Figure S2 displays the results for the PL configurational entropy, S_{PL}, for which we observe a slight but not significant, difference in results between the PI and vacuum systems.

The results for this PL subsets imply it is valid to develop the simulation procedure making use of simulating PL molecules in vacuum. This enables us to significantly reduce computational cost.

PL Miscibility

The following section displays example snapshots of miscible and immiscible PL topologies in a PI melt using the bead and spring model described in the main text. The concentration of PL molecules is fixed at approximately 5 *phr*. The PI melt has been removed from the visualisation. Figure S3(a) displays a system where $\zeta \approx 1.5$, which is typical of a system that displays miscible PL behaviour. In contrast, S3(b) shows a system with partial PL clusters formed and $\zeta \approx 2.7$, which we conservatively choose as a cut-off value to distinguish between miscible and immiscible PLs. The third image, S3(c), shows a system which has formed large clusters and, as such, has a significantly larger ζ value, $\zeta \approx 69.70$. Figure S4 shows an example of miscible (red) and immiscible (blue) PL behaviour in terms of the miscibility parameter, ζ. Both systems begin with PL molecules evenly dispersed throughout the simulation box. PL 10B-3S-0.2-r (miscible) remains fluctuating around a value of $\zeta = 1.7$ across the trajectory, whereas PL 10B-7S-0.4-r (immiscible) shows a rising value of ζ over approximately 5 μs which corresponds to an increase in PL clustering.

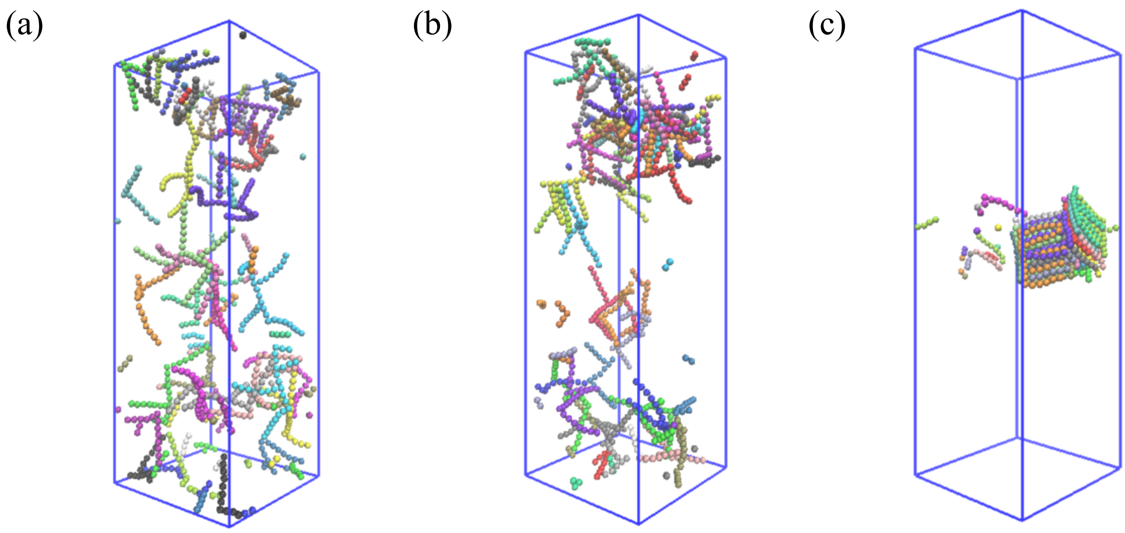

Figure S3: Snapshots of PL molecules in the PL/PI (PI not pictured) systems. The PLs depicted are **(a)** 10B-7S-0.3-r, **(b)** 10B-7S-0.4-r and **(c)** 10B-6S-0.5-r. The approximate ζ values are **(a)** 1.5, **(b)** 2.7 and **(c)** 69.70. Each PL molecule is independently coloured. From this, an increase in PL agglomeration can be observed from **(a)**-**(c)**.

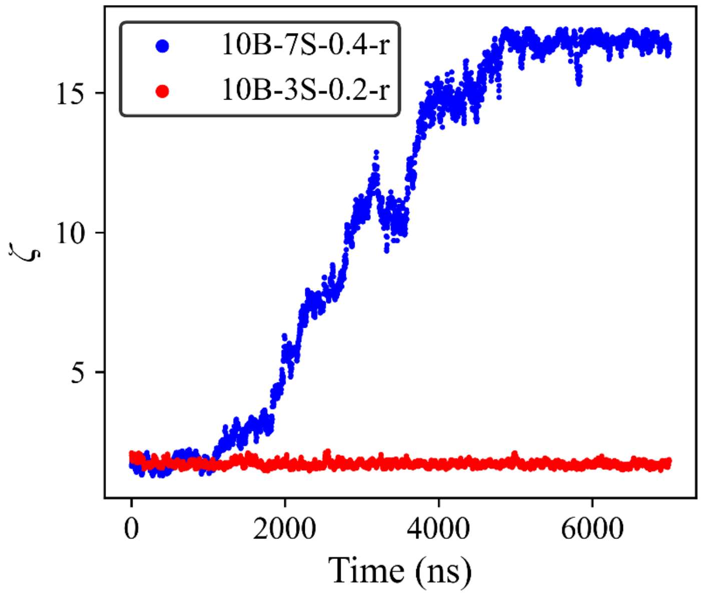

Figure S4: Miscibility factor, ζ, for PL 10B-3S-0.2-f (red) and 10B-7S-0.4-r (blue) against simulation time.

PL/PL System Equilibration

The equilibration of the PL/PL simulations which are used to calculate the PL configurational entropy of PLs in infinite clusters, $S_{PL/PL}$, is identified by calculating the probability density associated with Equation (11) with the time averaged PL end-to-end distance distribution over 600 ns blocks. We also average over each PL in the system for good statistics. We find this is sufficient time for $S_{PL/PL}$ to reach a plateau, which takes approximately 8 μs for the systems with rod-like PLs, as displayed in Figure S5. The other, flexible PL, systems have a significantly shorter equilibration period due to the less rigid nature of the constraints on their bond angles.

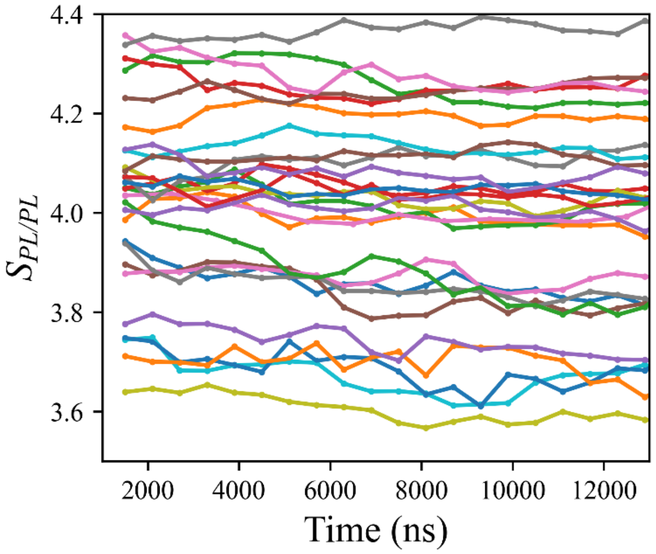

Figure S5: Plot showing trends in the equilibration periods of PL/PL simulations with rod-like PL topologies. Each colour represents a different PL topology and results for $S_{PL/PL}$ are extracted after an 8 μs time period.

Further Flexible PL Simulations

We performed simulations to calculate each descriptor for a further 3 flexible PLs; in order to verify that our procedure is unaffected by the placement of side chains on the first or last beads of the PL 'backbone', such that the molecules retain a more 'brush-like' shape comparable with their rod-like counterparts. The PLs simulated are depicted in Figure S6 and corresponding results for the R_g^2, c and S_{agg} descriptors are displayed in Figure S7.

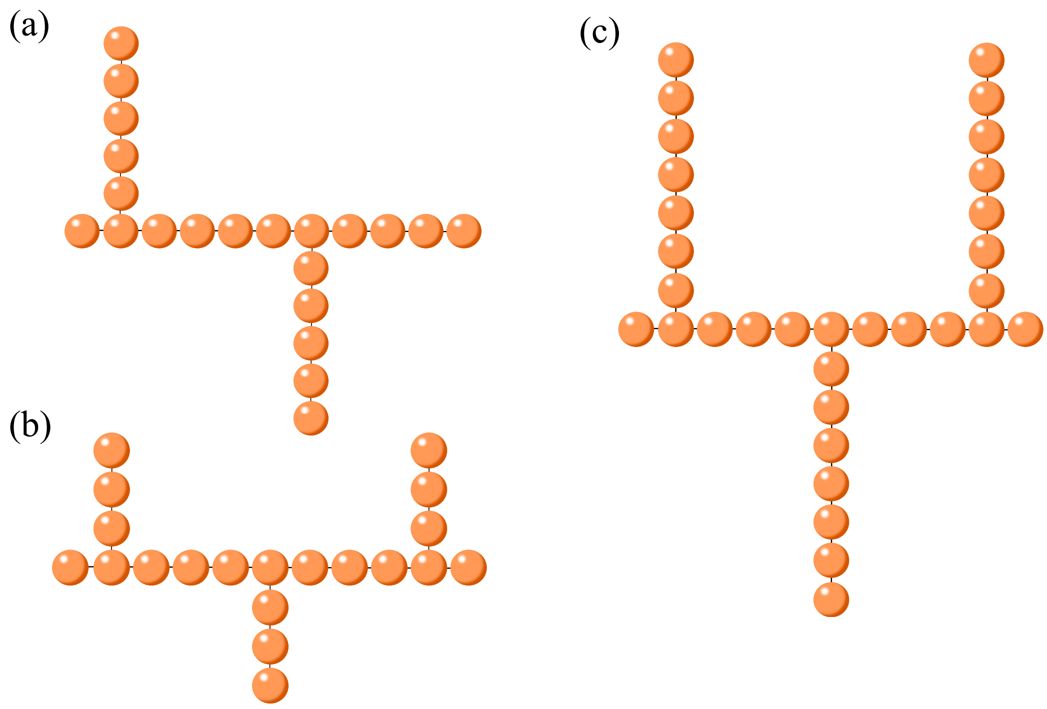

Figure S6: Schematic representations of the 3 additional flexible PLs simulated. Each backbone contains 11 beads and side chain lengths and frequencies were chosen to approximately reproduce the 'brush-like' shape present in the rod-like PLs displayed in **Figure 1(a)**, **(c)** and **(f)** of the main text.

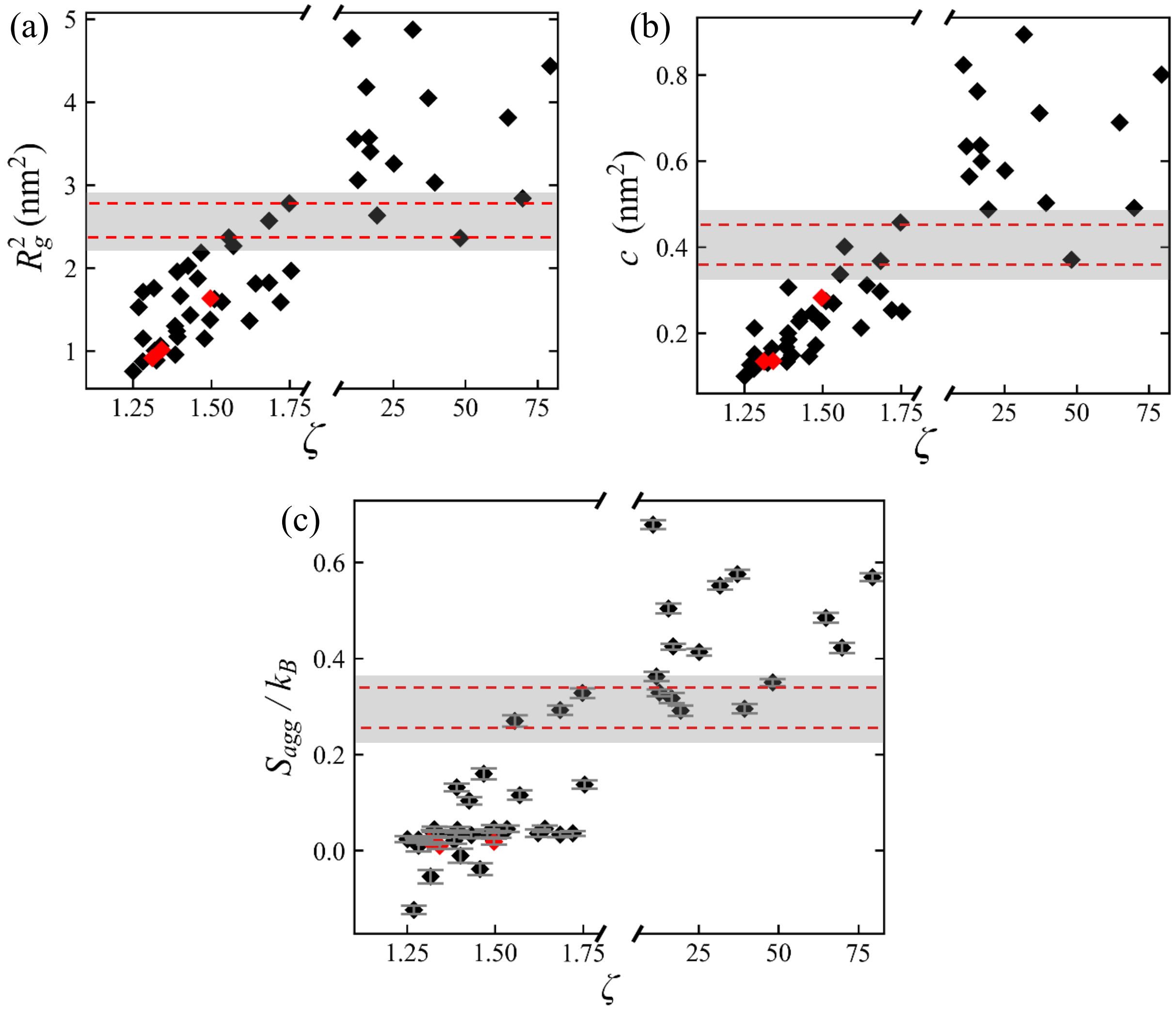

Figure S7: Plots of **(a)** R_g^2, **(b)** c and **(c)** S_{agg} against miscibility parameter, ζ, for the 48 PLs in this work, along with those displayed in Figure S8 (red).

The results are in line with our previous findings that flexible PLs are miscible within the PI matrix and their descriptor values correlate with ζ accordingly, regardless of the placement of their side chains.
